# Simplifying supplementation in MSUD: tolerance and acceptability of liquid valine and isoleucine supplements in maple syrup urine disease

**DOI:** 10.1186/s13023-026-04223-7

**Published:** 2026-04-02

**Authors:** Martina Tosi, Anne Daly, Sharon Evans, Alex Pinto, Catherine Ashmore, Anita MacDonald

**Affiliations:** 1 Department of Health Sciences, University of Milan, Milan, Italy; 2 Birmingham Children’s Hospital, Steelhouse Lane, Birmingham, B4 6 NH UK

**Keywords:** Maple syrup urine disease, Valine, Isoleucine, Leucine, Supplements

## Abstract

**Introduction:**

Maple Syrup Urine Disease (MSUD) is a rare metabolic disorder requiring lifelong restriction of branched-chain amino acids (BCAAs), and targeted supplementation with valine and isoleucine to maintain metabolic stability.

**Methods:**

A prospective, open-label, observational study was conducted over 56 days in 5 paediatric patients mean age 8.8 years (range 3–14) with classical MSUD. In Phase 1 (Days 0–28), participants received the ready to use (RTU) liquid valine formulation, while continuing their usual powdered isoleucine supplement. In Phase 2 (Days 29–56), participants returned to powdered valine and changed to the ready to use RTU liquid isoleucine supplement. Weekly plasma BCAAs were monitored. Acceptability, gastrointestinal tolerance, adherence, and dietary intake were assessed through questionnaires and food diaries.

**Results:**

RTU liquid valine and isoleucine supplements were well tolerated and accepted by all participants. Plasma leucine decreased when taking both liquid valine (mean leucine 253 µmol/L) and isoleucine (mean leucine 219 µmol/L) compared to baseline (mean leucine 356 µmol/L). Required doses of valine and isoleucine decreased by 30% and 18%, respectively, suggesting improved adherence or enhanced efficacy. Palatability and ease of use were rated more favourably for the RTU products, with no adverse effects or adherence issues reported. Dietary intake remained stable throughout the study.

**Discussion:**

These preliminary findings suggest that RTU valine and isoleucine formulations may offer potential advantages in MSUD management, simplifying administration, and enhancing user satisfaction. While limited by sample size and short-term evaluation, their practicality and patient-centered design warrant further investigation in larger, more diverse populations to establish long-term efficacy and broader applicability.

**Supplementary Information:**

The online version contains supplementary material available at 10.1186/s13023-026-04223-7.

Maple syrup urine disease (MSUD) is a rare inherited disorder of branched chain keto acid dehydrogenase (BCKDH), a mitochondrial multienzyme complex. This enzymatic defect results in the accumulation of branched-chain amino acids (BCAAs) in the blood, particularly leucine, and their corresponding keto acids. A hallmark feature of MSUD is the excretion of BCAAs in the urine, which emits a characteristic sweet, maple syrup-like odour. High levels of leucine lead to leucinosis, while the accumulation of its keto acid, α-ketoisocaproic acid (α-KIC), is considered responsible for disrupting cerebral aerobic metabolism. This may contribute to metabolic encephalopathy, neuropathological abnormalities, and potentially fatal cerebral oedema [1–3].

Five clinical phenotypes of MSUD have been described but the classical and intermediate forms are the most common. Classical MSUD, which is associated with less than 2–3% residual enzyme activity, accounts for approximately 50–75% of all cases [4]. The estimated global prevalence of MSUD is around 1 in 185,000 live births, but in the UK, the incidence is approximately 1 in 100,000 [5]. Since 2012, the UK has included MSUD in its national newborn screening programme [6]. Classical MSUD presents in the neonatal period with symptoms of ketonuria, a characteristic sweet maple syrup odour, irritability, poor feeding and vomiting. If untreated, it may lead to lethargy, seizures, cerebral oedema, encephalopathy, coma, and potentially death [7]. Without timely and effective treatment in the early postnatal period, prolonged high plasma leucine exceeding 1000 µmol/L is associated with irreversible brain injury and poor neurodevelopmental outcomes.

The BCAAs valine, leucine and isoleucine are indispensable amino acids and cannot be synthesised endogenously. They comprise approximately 35% of indispensable muscle proteins and 40% of total amino acids required by the human body [8]. Unlike most amino acids which are degraded in the liver, BCAAs are largely catabolised by extrahepatic tissues such as muscle, adipose tissue, kidneys and the brain. A deficiency in BCKDH impairs the oxidation of BCAAs leading to a disturbance in the concentration ratio of leucine, valine and isoleucine. Under normal physiological conditions, the isoleucine: leucine and leucine: valine ratios are approximately 0.5 µM and 2 µM, respectively [7]. A disturbance in this ratio can adversely affect the uptake of these essential amino acids by both the brain and muscle tissue. Leucine, isoleucine and valine share the same large neutral amino acid transport system (LAT 1) allowing entry across the blood brain barrier, and these BCAAs and are in constant competition with each other. Thus, inadequate supplementation of valine and isoleucine reduces this competition allowing a greater influx of leucine leading to reduced brain protein synthesis increasing cerebral damage. Additionally, none of the BCAAs are endogenously synthesized hence maintaining an adequate and balanced dietary intake of all three BCAAs is crucial to prevent both deficiency and excess [9, 10].

In classical MSUD, dietary treatment primarily leucine restriction, prevents neurotoxicity. However, this approach also reduces the dietary intake of valine and isoleucine, disrupting the BCAA balance. When the valine: leucine molar ratio decreases below 2, leucine transport across the blood–brain barrier can dominate, potentially leading to valine deficiency [11]. Although isoleucine is less susceptible to transport competition, maintaining its adequacy remains of equal importance. Valine deficiency has been associated with hypomyelination and skin lesions [7]. Therefore, frequent biochemical monitoring is essential [10], with careful maintenance of leucine within a narrow therapeutic target range of 100–300 µmol/L recommended in the UK. In addition, supplementation with valine and isoleucine, commonly starting at 50–100 mg/day, is necessary, aiming for plasma target levels between 200 and 400 µmol/L [8, 12]. This judicious supplementation avoids rate-limiting effects on protein synthesis and anabolic processes [13]. Given the variability in metabolic tolerance and individual response, valine and isoleucine dosing must be tailored through bespoke measurement and ongoing adjustment, guided by regular biochemical monitoring and clinical assessment.

Supplementation of valine and isoleucine is also an essential part of illness management in MSUD. During illness, individuals are at high risk of metabolic decompensation due to increased muscle breakdown and a rapid increase in plasma leucine levels, which can lead to lethargy and encephalopathy. Immediate and aggressive intervention is essential to prevent catabolism. This includes administration of a high-energy, high-carbohydrate emergency regimen, including a BCAA-free amino acid formula, glucose polymer and isoleucine and valine supplements [10]. Providing sufficient isoleucine and valine during acute illness supports protein synthesis and promotes more effective reduction in plasma leucine levels. Without adequate supplementation, leucine clearance is impaired, increasing the risk of prolonged decompensation. Therefore, isoleucine and valine should be included in all MSUD emergency feeds.

Valine and isoleucine supplementation presents practical challenges due to limited availability of suitable preparations. In the UK, two powdered forms of single amino acid supplements are available. These are (1) pre-measured powdered sachets (Valine 50 and 1000; Isoleucine 50 and 1000, Vitaflo International) providing either 50 mg or 1000 mg of valine or isoleucine per sachet, with added carbohydrate (3.8 g per 4 g sachet) and (2) bulk powder tubs (Nutricia Ltd), containing 100 g of valine or isoleucine without added carbohydrate. They require precise measurement due to the small doses typically prescribed. In regions such as Iran, where individual valine and isoleucine supplements are unavailable, dietitians have adapted emergency protocols using leucine-free formulations that contain valine and isoleucine to manage acute MSUD episodes [14]. Furthermore, a novel approach using 3D printing technology has also been explored by Goyanes et al. [15] in a prospective crossover study. Two isoleucine formulations were compared: manually prepared capsules and chewable printlets that were bespoke, flavoured, and coloured dosage forms produced via 3D printing. The printlets demonstrated improved pharmacokinetic consistency, with isoleucine levels showing reduced variability and closer alignment with target plasma levels. They also had enhanced acceptability, particularly with children, due to their sensory appeal and ease of administration. In Germany, tablet preparations of valine and isoleucine (Metax Ltd) are available, though their use is generally restricted to older children and adults capable of swallowing tablets.

Powdered isoleucine and valine supplements mix easily with powdered protein substitutes. However, for patients prescribed liquid BCAA-free protein substitutes, powdered valine and isoleucine are less practical, they must be prepared separately, often mixed with another liquid, increasing both preparation burden and the risk of incomplete intake if the supplements are not fully consumed. Although typically divided into three daily doses, optimal dosing schedules for valine and isoleucine have not been studied, and adherence to these supplements remains unreported.

Ready-to-use (RTU) flavoured liquid formulations of valine (50 mg/mL) and isoleucine (25 mg/mL), (Meta Healthcare Ltd) were evaluated in a prospective observational study involving five children with classical MSUD. Designed for use alongside liquid protein substitutes, the preparations aimed to address the limitations of powdered supplements in patients with MSUD. The study assessed the efficacy, acceptability, and tolerance of the RTU liquid formulations over a 56-day period, with participants replacing their usual powdered valine and isoleucine supplements in two consecutive 28-day phases.

## Materials and methods

### Subject selection

The inclusion criteria included children aged 0–16 years with a confirmed diagnosis of classical MSUD. They had well-controlled plasma leucine maintained within the target therapeutic range for at least 4 weeks prior to enrolment. Participants and their caregivers were required to adhere with study procedures and provide informed consent or assent. All children took powdered valine and isoleucine supplements pre-baseline. Exclusion criteria included individuals with non-classical MSUD, those non-adherent with dietary management, or with plasma leucine above target therapeutic recommendations. Additional exclusion criteria included children who had undergone liver transplantation or had any comorbidities or acute illnesses likely to affect study outcomes.

### Study products

Two RTU liquid supplements of valine and isoleucine (Meta Healthcare Ltd), were evaluated.

Both RTU liquids were clear, straw-coloured, strawberry-flavoured, and free from carbohydrate. They contained saccharin (artificial sweetener), strawberry flavouring, thickeners, and preservatives, as outlined in Table 2. The RTU valine solution was a 50 mg/mL formula and the isoleucine solution a 25 mg/mL formula.

The study products were supplied in 100 mL bottles and stored at temperatures below 25 °C after opening. A dosing syringe was provided to ensure precise administration. Individual dosages were determined by the clinical dietitians, based on each participant’s existing powdered supplement regimen and plasma valine and isoleucine levels. Both RTU liquid formulations shared the same excipients, as listed in Table 1, differing only in the active amino acid: valine or isoleucine.

**Table 1:** Compares the RTU formulations with existing powdered products

	Vitaflo Ltd		Nutricia Ltd	Vitaflo Ltd		Nutricia Ltd	Meta Healthcare Ltd	
**Name**	Valine 50	Valine 1000	L-Valine	Isoleucine 50	Isoleucine 1000	L-Isoleucine	L-Valine 50 mg/1mL	L-Isoleucine 25 mg/1mL
**Presentation** Energy kcal (kJ) Carbohydrate (g) Sugars (g) Fibre (g) Fats (g) Protein Equivalent(g) **L-Valine (mg)** **L-Isoleucine (m** **g) ** **Sodium(g)**	**4 g sachet**		**100 g/tub**	**4 g sachet**		**100 g/ tub**	**100mL**	**100mL**
	15 3.8 0 0 0 0.04 **50** 0 -	15 2.9 0 0 0 0.8 **1000** 0 -	338 (1438) 0 0 0 0 84.6 **100,000** 0 -	15 3.8 0 0 0 0.04 0 **50** -	15 2.9 0 0 0 0.8 0 **1000**	345 (1476) 0 0 0 0 86.3 0 **100,000** -	10 (44) 0 0 0 0 1.7 **5000** 0 0.3	10 (44) 0 0 0 0 1.7 0 **2500** 0
**Ingredients**	Dried Glucose Syrup, L-Valine		L-Valine	Dried Glucose Syrup L-Isoleucine		L-Isoleucine	Excipients: Sodium methyl/ propyl parabens, sodium saccharin, citric acid anhydrous, disodium hydrogen orthophosphate, natrosol 25OHX pharma, strawberry flavour, water	

### Study design

This was a 56-day, prospective, open-label observational study. The study followed a two-phase, crossover design in which each participant served as their own control (Table 2).

Phase 1 (Days 0–28): Participants replaced their usual powdered valine supplement with the RTU liquid valine formulation, while continuing with isoleucine powder.

Phase 2 (Days 29–56): Participants replaced their usual powdered isoleucine supplement with the RTU liquid isoleucine formulation but returned to the valine powder.

**Table 2:** Representation of the study design

MSUD *n*=5 subjects				
Phase 1		Phase 2		
Day 0 - start of phase 1	**Day 28 - end of phase 1**	**Day 29 - start of phase 2**		**Day 56 - end of phase 2**
• Stop valine powder • Start valine liquid • Continue isoleucine powder	• Stop valine liquid • Recommence valine powder	• Stop isoleucine powder • Start liquid isoleucine • Continue valine powder		• Stop liquid isoleucine • Recommence powder valine and isoleucine
• 3-day dietary intake • Height and weight • Gastrointestinal symptom daily dairy • Evaluation of valine powder acceptability • Evaluation of valine powder tolerance	• 3-day dietary intake • Height and weight • Gastrointestinal symptom daily dairy • Evaluation of valine liquid acceptability • Evaluation of valine liquid tolerance	• 3-day dietary intake • Height and weight • Gastrointestinal symptom daily dairy • Evaluation of isoleucine powder acceptability • Evaluation of isoleucine powder tolerance		• 3-day dietary intake • Height and weight • Gastrointestinal symptom daily dairy • Evaluation of isoleucine liquid acceptability • Evaluation of isoleucine liquid tolerance

Weekly plasma valine, isoleucine and leucine monitoring: week 0–8

Dietary assessments, and anthropometry (weight, height and BMI) were collected at three time points: baseline, end of phase 1 and phase 2 (days 0, 28 and 56). Anthropometry was measured by two research dietitians (AM, AD), height was measured using a Seca stadiometer to the nearest 0.1 cm and weight on calibrated digital scales to the nearest 0.1 g (Seca Medical Measuring Systems and Scales, Birmingham UK – Model 875). Medical history, oral medications and any treatment related adverse reactions (TRAR) were recorded. Structured 3-day food records were completed with caregiver support to enhance accuracy and comprehensive documentation. Data was collected on leucine, protein substitute, and total protein intake. Records were subsequently reviewed and analyzed by trained metabolic dietitians.

Caregivers maintained a structured daily adherence diary throughout the intervention period, documenting intake of valine and isoleucine supplements. A non-validated, psychometric Likert scale (0–5) was used to capture multiple dimensions of tolerability and adherence, with 0 indicating a negative response and 5 a positive response (Supplementary questionnaire 1). 

Caregivers also maintained a daily gastrointestinal symptoms record. Nausea, bloating, flatulence, and vomiting were recorded using the same Likert scale, providing a quantitative measure of symptom severity (Supplementary questionnaire 2). Tolerance of the liquid supplement was also assessed with the same scale, where 0 indicated complete intolerance and 5 indicated excellent tolerability (Supplementary questionnaire 3).

Adherence was defined as the extent to which the supplement was taken at the prescribed dosage and frequency, and the amount that was taken each day was recorded. 

### Biochemical monitoring

One month preceding the study, weekly liquid BCAA blood samples were collected to assess metabolic stability. During the intervention, weekly plasma concentrations of valine, isoleucine and leucine were collected by the parents at home, sent to the hospital laboratory where they were analysed to monitor metabolic control whilst taking the RTU liquid valine and isoleucine formulations. Liquid blood samples were collected in Starsted lithium heparin tubes and separated by centrifuge. Plasma samples were deproteinised via the addition of sulfosalicylic acid and internal standard, S-(2-Aminoethyl)-L-cysteine hydrochloride (AEC) (Sigma A2636), frozen and then centrifuged. Amino acid analysis was performed by lithium citrate, ion exchange chromatography with post-column ninhydrin derivatisation using a Biochrom30 + HPLC analyser.

### Product acceptability and tolerance

At the beginning and end of each intervention phase, participants and their caregivers completed a structured, but non-validated questionnaire comparing the RTU liquid valine and isoleucine supplements with their usual powdered versions. The questionnaire assessed key factors such as adherence, smell, taste, palatability, aftertaste, and ease of use. Responses were rated on a five-point Likert scale: Great (5), Good (4), OK (3), Bad (2), and Terrible (1), with scores converted to numerical values for analysis (Supplementary Tables 1 to 3). These questionnaires were selected in accordance with guidance from the UK Advisory Committee for Borderline Substances (ACBS), which governs the regulatory framework for the prescription of new dietetic products. As part of this framework, the use of standardised questionnaires is stipulated by the ACBS and our methodology was designed to comply with these national requirements.

### Statistical analysis

No formal sample size calculations were performed, as all eligible patients who met the inclusion criteria were recruited for this study. Given the limited sample size, statistical comparisons were not feasible; results are therefore presented descriptively and should be interpreted as exploratory. Quantitative data and continuous variables were summarized using mean ± standard deviation (SD), while categorical variables were presented as absolute counts and percentages.

### Ethical approval

The study ‘Valine 50 mg/mL and isoleucine 25 mg/mL oral solutions for Maple Syrup Urine Disease (MSUD) – Acceptability and Tolerance Study’ was given favourable ethical approval by the West Midland, South Birmingham Research Ethical Committee (REC reference, 24/WM/0171; IRAS project ID, 324480; ClinicalTrials.gov ID NCT06581991). The study was conducted in adherence with the guidelines of the Local Ethical Committee and in accordance with the ethical standards of the 2024 Helsinki Declaration. Written informed consent and assent for the use of anonymized data was obtained from each participant’s parent or legal guardian.

## Results

### Subjects

Five participants with classical MSUD, three females and two males, median age 8.8 years (range 3 to 14 years) were recruited. Their ethnicity was British Pakistani (*n* = 4) and British Bangladeshi (*n* = 1) One participant was identified by newborn screening, *n* = 2 diagnosed by clinical symptoms in the neonatal period, and *n* = 2 by sibling screening.

### Product administration

At baseline, all participants were using powdered valine or isoleucine supplements, either mixed into their BCAA-free powdered protein substitute or, for those on liquid protein substitutes, added to fruit squash. Upon transition to the RTU valine and isoleucine formulations, four participants accepted the supplements directly via oral syringe. In one case, for a three-year-old participant, the liquid supplements were mixed with the powdered protein substitute, replicating the administration method previously used for powdered valine and isoleucine. This participant declined to taste the liquid supplements via an oral syringe. It remains unclear whether this refusal was due to neophobia associated with a novel product or an aversion to the syringe delivery method, potentially linked to prior antibiotic administration. As palatability could not be directly assessed in this case, the participant’s score was excluded from the analysis (see Supplementary Table 1). 

### Anthropometric measurements

Weight, height and BMI were consistent throughout the study. Table 3 describes the subjects gender ethnicity, age and anthropometry.

**Table 3:** Demographic and anthropometric data for MSUD subjects

Subjects	Gender	Age	Ethnicity	Day 0 start phase 1						Day 28 end of phase 1/ start phase 2						Day 59 study end phase 2					
				Height (cm)	z-score	Weight (kg)	z-score	BMI	z-score	Height (cm)	z-score	Weight (kg)	z-score	BMI	z-score	Height (cm)	z-score	Weight (kg)	z-score	BMI	z-score
**1**	F	12	British Pakistani	146.4	-0.7	56.5	+ 1.5	26.3	+ 2.2	146.7	-0.7	57.8	+ 1.6	26.8	+ 2.3	147	-0.7	57.3	+ 1.5	26.5	+ 2.2
**2**	M	14	British Pakistani	160.5	-1.5	83.5	+ 1.9	32.4	+ 2.9	160	-1.6	82.7	+ 1.8	32.3	+ 2.8	161.4	-1.5	83	+ 1.8	31.9	+ 2.8
**3**	M	3	British Pakistani	93.5	-1.2	15.9	+ 0.2	18.2	+ 1.5	95.5	-0.8	15.6	0	17.1	+ 0.8	96	-0.8	14.4	-0.7	15.6	-0.2
**4**	F	8	British Pakistani	119.7	-1.8	35.2	+ 1.3	24.6	+ 2.6	120.4	-1.8	35.8	+ 1.3	24.7	+ 2.6	120.6	-1.9	35.8	+ 1.3	24.6	+ 2.7
**5**	F	7	British Bangladeshi	123.2	-0.3	24.4	0	16.1	+ 0.1	123.4	-0.5	24.6	-0.2	16.1	+ 0.1	123.7	-0.5	24.7	-0.2	16.1	+ 0.1

### Dietary intake

The amount of natural protein and protein equivalent form BCAA-free protein substitute is presented in Table 4. The natural protein and protein equivalent intake from protein substitute remained consistent over the intervention period, with the exception of one participant (3) whose natural protein intake increased by 2 g/day due to lower plasma leucine levels.

**Table 4:** Description of the daily amount of natural protein and protein equivalent for each patient

Subjects	Natural protein g/day (100 mg leucine)	Protein equivalent from protein substitute (g/day)	Type of protein substitute	Total protein (g/day)
1	6	80	Ready-to-drink	86
2	7	75	Gel	82
3*	5 to 7	45	Spoonable gel	50–52
4	4	60	Ready-to-drink	64
5	5	60	Ready-to-drink	65

* Increase in dietary leucine over the study period due to lower plasma leucine

### Clinical assessment

During Phase 2 of the study, one participant experienced an unrelated intercurrent illness. This was a chest infection requiring a short course of oral antibiotics. The child was managed at home and did not require hospital admission. This event occurred during the final days of isoleucine supplementation, minimizing any potential carryover effects into subsequent phases. No protocol deviations or adverse events related to study interventions were reported.

### Biochemical assessment

There was a noticeable decrease in blood leucine concentrations when using RTU liquid valine and isoleucine compared to powdered preparations as shown in Table 5.

**Table 5:** Mean and range of weekly blood valine, isoleucine and leucine concentrations pre-study and when taking liquid valine and isoleucine supplements

Subject	Time of collection	Mean blood results and range (µmol/L) and standard deviation					
		Leucine	SD	Isoleucine	SD	Valine	SD
**1**	Pre-study	254 (185–370)	80.86	243 (212–266)	23.69	442 (412–480)	29.98
	On liquid Valine	251 (129–351)	102.7	251 (171–309)	62.02	513 (361–703)	149.2
	On liquid Isoleucine	238 (223–264)	22.85	266 (220–312)	46.00	487 (428–594)	92.57
**2**	Pre-study	716 (167–1278) *	518.3	381 (262–485)	123.3	516 (428–594)	195.7
	On liquid Valine	199 (113–378)	121.7	284 (248–319)	34.32	443 (291–555)	121.4
	On liquid Isoleucine	106 (88–130)	19.24	239 (223–319)	41.67	610 (516–668)	76.95
**3**	Pre-study	284 (138–590)	211.9	312 (155–459)	130.7	289 (173–363)	86.64
	On liquid Valine	272 (222–329)	53.54	313 (237–403)	72.58	362 (331–385)	25.16
	On liquid Isoleucine	324 (173–575)	180.5	366 (120–460)	156.2	324 (167–575) ^**§**^	180.5
**4**	Pre-study	313 (237–401)	68.38	279 (223–379)	69.48	439 (381–515)	68.34
	On liquid Valine	195 (153–231)	37.37	199 (168–280)	54.05	502 (391–592)	92.27
	On liquid Isoleucine	217 (171–268)	51.99	287 (253 − 248)	105.3	354 (296–403)	47.49
**5**	Pre-study	214 (150–268)	48.84	286 (232–364)	62.68	443 (312–590)	114.6
	On liquid Valine	349 (158–684)	232.3	183 (116–276)	67.99	301 (245–449)	99.21
	On liquid Isoleucine	211(170–284)	50.57	342 (183–479)	125.3	395 (327–461)	56.18
**Mean (range) values**	Pre-study	356 (214–716)	204.5	300 (243–381)	51.46	426 (289–516)	83.04
	On liquid Valine	253 (195–349)	62.97	246 (183–313)	55.08	424 (301–513)	91.29
	On liquid Isoleucine	219 (106–324)	77.82	292 (226–366)	61.58	434 (324–610)	116.0

* Subject number 2, infection pre study; ^§^ subject number 3, infection on antibiotics; SD standard deviation

### Change in valine and isoleucine doses (Table 6)

**Valine**: at baseline, the mean intake of powdered valine was 150 mg/day (range: 100–200 mg/day). With the study RTU formulation, this decreased to 100 mg/day (range: 50–150 mg/day), representing a 30% reduction. Despite the lower dose of valine, blood valine levels remained within target therapeutic recommendations. Mean blood valine levels prior to the study were 426 µmol/L (range: 289–516 µmol/L) and remained stable during the intervention at 424 µmol/L (range: 301–513 µmol/L).

**Isoleucine**: at baseline, the mean intake of isoleucine from the powdered supplement was 200 mg/day (range100–300 mg/day). With the study RTU formulation, this decreased to 160 mg/day (range: 100–300 mg/day), reflecting a 18% reduction. Despite the lower dose of RTU isoleucine, blood isoleucine levels remained stable, and was 300 µmol/L (range: 243–381 µmol/L) at baseline and 292 µmol/L (range: 226–366 µmol/L) during the intervention.

**Leucine**: Blood leucine levels remained stable throughout both study phases. There was a reduction in blood leucine observed during administration of the RTU valine and isoleucine supplements. Prior to the intervention, the mean blood leucine level was 356 µmol/L (range: 214–719 µmol/L), decreasing to 253 µmol/L (range: 195–349 µmol/L) during the RTU valine phase, and to 219 µmol/L (range: 106–324 µmol/L) during the RTU isoleucine phase.

**Table 6:** Changes in daily doses of valine and isoleucine supplements from baseline and over the intervention period

Subject	Valine supplementation				% Reduction	Isoleucine supplementation				% Reduction
	Pre study powder daily dose (mg Val)	Start day 0	End phase 1 day 28	Liquid daily dose (mg Val)		Pre study powder daily dose (mg Iso)	Start day 29	End phase 2 day 59	Liquid daily dose (mg Iso)	
**1**	150 mg	3 ml 2 doses	2 ml 2 doses	100 mg	-33%	200 mg	8 ml 2 doses	6 ml 2 doses	150 mg	-25%
**2**	200 mg	4 ml 3 doses	2 ml 2 doses	100 mg	-50%	250 mg	10 ml 3 doses	5 ml 3 doses	125 mg	-50%
**3**	150 mg	3 ml 3 doses	3 ml 3 doses	150 mg	0%	150 mg	6 ml 3 doses	5 ml 3 doses	125 mg	-17%
**4**	150 mg	3 ml 2 doses	1 ml 1 dose	50 mg	-67%	300 mg	12 ml 2 doses	12 ml 2 doses	300 mg	0%
**5**	100 mg	2 ml 1 dose	2 ml 1 dose	100 mg	0%	100 mg	4mL 1 dose	4mL 1 dose	100 mg	0%
**Mean**	**150 mg**	**3 ml**	**2 ml**	**100 mg**	**30%**	**200 mg**	**8 ml**	**6.4 ml**	**160 mg**	**18%**

Valine 50 mg / 1mL, Isoleucine 25 mg/1 mL

The RTU valine and isoleucine supplements contained a minimal amount of sodium (2.6 mg/mL). Supplemental Table 4 shows values of sodium intake compared to UK reference guidelines [16]. Compared to the powdered preparation there was an improvement in palatability, acceptability, and ease of use with the RTU products and no significant changes in gastrointestinal symptoms were reported (Supplemental Tables 1–3).

## Discussion

This is the first study to evaluate the use of newly developed RTU liquid valine and isoleucine supplements in children with classical MSUD. Prior to the availability of these formulations, suitable UK products were limited to powdered preparations, either in pre-measured sachets or requiring manual measurement using scoops or scales. Valine and isoleucine are essential components of MSUD management: they competitively inhibit leucine transport into the central nervous system and support protein anabolism by preventing acute deficiencies, both of which are rate-limiting for leucine metabolism. Prolonged deficiencies of one or more branched-chain amino acids, whether due to excessive dietary restriction or inadequate supplementation, may result in poor growth and anaemia, compromised mucosal integrity, and global developmental delay [17, 18].

During intercurrent illness, maintaining endogenous protein synthesis and preventing amino acid imbalance is critical. In infants at risk of metabolic decompensation, particularly those requiring intensive care or haemofiltration, leucine free amino acids that include valine and isoleucine, are vital to promote anabolism. Emergency settings often necessitate fluid restriction, and powdered preparations may increase feed osmolarity due to added carbohydrate content, potentially compromising gastrointestinal tolerance. The RTU valine and isoleucine liquid supplements used in this study were carbohydrate-free, easily measured, and reduced the risk of preparation errors in both hospital and home environments, offering a practical alternative during periods of clinical instability.

Both RTU valine and isoleucine supplements were well tolerated and generally preferred over the powdered formulations. Notably, during the 8-week intervention, plasma leucine levels declined following the introduction of both the RTU liquid valine and isoleucine. Although the small sample size precluded statistical significance testing, the observed reductions in blood leucine levels were potentially useful: leucine decreased from 356 µmol/L to 253 µmol/L with RTU liquid valine, and to 219 µmol/L with liquid isoleucine. Variability in blood leucine concentrations was observed within subjects across the study phases. While the mean leucine concentration decreased from baseline during the RTU supplementation period, this trend was not consistent across all participants, specifically, subjects 3 and 5 reflecting the variability that is characteristic of classical MSUD. Without kinetic studies comparing powder and liquid preparations it remains uncertain whether lowering of blood leucine reflects the property of the liquid formulations or improved adherence facilitated by their convenience. The RTU format eliminates the need for reconstitution, possibly enhancing consistency of administration and, potentially efficacy. To determine causality, a controlled, randomized study incorporating objective adherence measures is required. Although premeasured sachets were convenient, issues with solubility and potential residue, either in the cup or sachet, may have contributed to underdosing, as previously reported by Gokmen et al. [19] Some older children also reported difficulty with the palatability of the powdered valine and isoleucine supplements, particularly when mixed with fruit squash. As this study evaluated RTU liquid valine and isoleucine in separate phases, further research is needed to assess outcomes when both are administered concurrently over a longer period.

An interesting and unexpected observation in this study was the reduction in valine and isoleucine dosages, by 30% and 18%, respectively, potentially indicating either improved efficacy, reduced wastage, enhanced adherence or a combination. Participants generally reported better palatability with the RTU liquid formulations, and they transitioned without difficulty, with caregivers noting no issues in administration. It is common in MSUD, for patients to display neophobia resisting any alterations in food and protein substitute preferences, so this seamless change was particularly noteworthy. Several participants highlighted a preference for the taste of the RTU liquid isoleucine, possibly due to its lower concentration (25 mg/mL) compared to valine (50 mg/mL). Although a few found the RTU liquid isoleucine slightly too sweet, all patients chose to continue with both new supplements beyond this short assessment, pointing to strong overall acceptance and positive user experience.

Participant adherence with the new RTU supplements was consistent, with all individuals taking them as prescribed throughout the study period. These findings confirm the broader perspectives of Morel et al. [20] who examined how individuals with rare diseases and their caregivers assess the trade-offs between treatment benefits and risks. Their research highlights the importance of integrating patient and caregiver preferences into therapeutic decision-making, especially for rare conditions where treatment options can be both limited and demanding. While not specific to MSUD, their conclusions underscore the significance of factors such as acceptability, ease of use, and patient-reported outcomes. The positive feedback on the palatability and practicality of the RTU liquid valine and isoleucine formulations observed in this study supports the value of patient-centered improvements in enhancing adherence and treatment satisfaction.

Nutritional intake, from both natural protein and protein substitutes, remained stable throughout the study, indicating that the change to the RTU liquid valine and isoleucine supplements did not impair dietary balance. Notably, one patient was able to increase natural protein intake without compromising metabolic control. The RTU liquid formulations were calorie-free, eliminating the need for adjustments in daily calculations.

The RTU valine and isoleucine formulations did contain a minimal amount of sodium 0.0026 g (2.6 mg/mL), derived from excipients. However, even at the highest intake, no participant consumed more than 0.034 g (34 mg/day). Given the UK guideline [16] that children aged 7–10 years should consume no more than 2 g (2000 mg) of sodium daily, and that most participants did not exceed 1 mg/day from these supplements, sodium intake was well within safe limits.

This study highlighted the potential application of RTU liquid single amino acid supplements in other inherited metabolic disorders where BCAA levels are commonly compromised. In urea cycle disorders (UCDs), BCAAs are often depleted in patients treated with nitrogen scavengers such as sodium phenylbutyrate or glycerol phenylbutyrate. These agents facilitate nitrogen excretion by converting excess nitrogen into phenylacetylglutamine (PAGN), which is then eliminated via the urine. Glutamine is the primary nitrogen donor for PAGN synthesis. Glutamine is made either from glutamate and ammonia or from BCAAs which donate nitrogen to alpha ketoglutarate making glutamate. The glutamate formed from BCAAs is used to synthesis glutamine allowing nitrogen to be donated for PAGN synthesis. The use of phenylbutyrate by using glutamine to excrete nitrogen, leads to BCAA depletion [21, 22]. There is also a growing but clinically debated interest in valine and isoleucine supplementation for individuals with methylmalonic acidaemia (MMA) and propionic acidaemia (PA). Several authors, including Manoli, van Vliet, and Myles [13, 23, 24], have raised concerns regarding disorder-specific protein substitutes for MMA and PA that are disproportionately high in leucine and deficient in valine and isoleucine. These imbalances in the BCAA profile have been associated with suboptimal growth, iatrogenic methionine deficiency, and a dermatological condition resembling acrodermatitis dysmetabolica. In such cases, targeted supplementation with individual valine and isoleucine preparations has been given to correct deficiencies and resolve associated skin lesions [25, 26]. Given the complex dietary regimens combined with pharmaceutical treatments in these conditions, RTU liquid valine and isoleucine supplements offer an alternative to powdered preparations.

The future development of a protein substitute for MSUD that incorporates baseline valine and isoleucine supplementation warrants exploration, despite its inherent complexity. Such a formulation could potentially deliver minimal safe intakes of valine and isoleucine, reducing reliance on separate supplementation and alleviating the treatment burden for families and patients. Emerging strategies, such as mathematical modelling of valine and isoleucine requirements across large MSUD cohorts, may offer valuable insights into optimal inclusion thresholds. However, interindividual variability, including growth stage, metabolic control, and catabolic episodes, remains a significant barrier to standardisation. As a result, the modular use of individual amino acid supplements is likely to remain essential, enabling precise and responsive adjustments tailored to each patient’s metabolic profile.

This study has several limitations. The small sample size precluded formal statistical analysis and limits the generalisability of the findings. Nonetheless, the observed reduction in valine and isoleucine dosing requirements, while maintaining blood concentrations within clinically acceptable ranges, remains clinically relevant and supports the practical utility of RTU valine and isoleucine formulations. As an observational study without randomisation or blinding, the design inherently limits causal inference and introduces potential bias. The absence of a comparator arm and the lack of a study phase in which both RTU supplements were administered concurrently further constrained the ability to assess combined effects. However, the split-dose methodology was intentionally selected to enable accurate assessment of metabolic responses to each individual intervention and to allow for a cautious, stepwise introduction, minimising the risk of metabolic instability. Following study completion, all participants transitioned to concurrent use of RTU valine and isoleucine supplements and have maintained metabolic stability, supporting the feasibility and safety of this approach in routine clinical practice.

Adherence was assessed using non-validated measures, which may have affected the reliability of the findings. The use of a non-validated Likert scale may have failed to capture subtle nuances in participant responses and limits comparability with other studies. Future randomised controlled trials with larger cohorts and validated adherence metrics are warranted to confirm these preliminary observations and to explain the mechanisms underlying improved biochemical control. However, MSUD is a rare and potentially life-limiting condition, with each specialist centre typically caring for a small number of patients. This significantly limits the feasibility of conducting large-scale, randomised studies and restricts opportunities to optimise study design and generate robust, generalisable evidence.

Although the findings were derived from a relatively homogeneous cohort, similar in ethnicity and geographic location, the successful administration of RTU liquid valine and isoleucine supplements suggests potential for broader clinical adoption. While all participants had classical MSUD with documented histories of restrictive eating behaviours, caregivers reported a seamless and error-free transition to the RTU formulations, highlighting their acceptability and ease of implementation in home settings. This reflects positively on both the usability and perceived effectiveness of the supplements, particularly in real-world settings where preparation burden and palatability often hinder adherence. The convenience and acceptability of RTU liquid valine and isoleucine supplements may offer an advance in the practical dietary management of MSUD, warranting further exploration in more diverse populations.

## Conclusions

In conclusion, this study supports the use of RTU liquid valine and isoleucine supplements in individuals with MSUD, demonstrating acceptability, good tolerance, and improved metabolic control. To strengthen these preliminary findings, future research involving larger, more diverse populations and extended follow-up periods is warranted. Such studies will help evaluate the long-term clinical outcomes and the potential impact on quality of life.

## Supplementary Information

Below is the link to the electronic supplementary material.

Supplementary Material 1: **Appendix A.1** Supplementary tables. Table 1 Acceptability review of liquid valine and isoleucine, Table 2 Gastrointestinal Symptom Diary, Table 3 Tolerance of new liquid valine and isoleucine compared to powdered preparations, Table 4 Sodium intake

## Data Availability

All data can be requested from the author.

## Abbreviations

MSUD: Maple Syrup Urine Disease
Val: valine
Iso: isoleucine
BCAA: branched chain amino acids
RTU: ready to use
