# Supplementary material for: Simplifying supplementation in MSUD: tolerance and acceptability of liquid valine and isoleucine supplements in maple syrup urine disease
Source: Orphanet J Rare Dis. 2026 Apr 2;21:124. doi: 10.1186/s13023-026-04223-7 (PMC13045159; doi:10.1186/s13023-026-04223-7)
Supplement: Supplementary file 1 — Supplementary Material 1: Appendix A.1 Supplementary tables. Table 1 Acceptability review of liquid valine and isoleucine, Table 2 Gastrointestinal Symptom Diary, Table 3 Tolerance of new liquid valine and isoleucine compared to powdered preparations, Table 4 Sodium intake [file 13023_2026_4223_MOESM1_ESM.docx]

**Appendix A1**

**Supplementary tables showing 1) psychometric Likert scale, 2) gastrointestinal symptoms, 3) tolerance and 4) sodium intake of the RTU liquid supplements**

**Supplementary table 1** Non validated psychometric Likert scale (0-5) used to capture acceptability to the valine and isoleucine RTU and powdered supplements

Likert scale ratings comparing powdered and liquid valine and isoleucine supplements (rounded to the nearest whole figure)

Great = 5, Good = 4, OK = 3, Bad = 2, and Terrible = 1

|  | **Valine**  **powder** | **Valine**  **liquid** | **Isoleucine**  **powder** | **Isoleucine**  **liquid** |
| --- | --- | --- | --- | --- |
| **Appearance** | 3 | 3**^§^** | 3 | 3**^§^** |
| **Smell** | 3 | 4 **^§^** | 4 | 4**^§^** |
| **Taste** | 3 | 4***^§^** | 3 | 3**^§^** |
| **Texture** | 2 | 4**^§^** | 2 | 4**^§^** |
| **Ease of mixing** | 2 | N/A | 2 | N/A |
| **Ease of taking** | 3 | 5**^§^** | 3 | 4**^§^** |
| **Aftertaste** | 3 | 3***^§^** | 3 | 4***^§^** |
| **Mean** | **3** | **4** | **3** | **4** |

*One participant noted that the taste of RTU valine and the aftertaste of both valine and isoleucine were not applicable, citing an inability to perceive any flavour, pleasant or unpleasant. § Subject 3 did not taste the product (powder or liquid) as this was added to the powdered protein substitute and has been excluded from the acceptability evaluation (appearance, smell, taste, texture and aftertaste for both powder and liquid products)

**Appearance** – what does the product look like

**Smell-** what does the product smell like

**Taste** – what does the product taste like

**Texture** – what is the mouth feel of the product (e.g. smooth, gritty, greasy)

**Easy of mixing** -how well does the product mix with liquids, powders

**Ease of taking**- how easy is it to take the product

**After taste-** is there an aftertaste when taking the product

**Supplementary table 2** summary of gastrointestinal symptoms

Every day the caregivers were asked: Did your child suffer from any of the following today

A number for each symptom was entered according to the following scale

None = 0, Mild = 1, Moderate = 2, Bad = 2, Severe = 4.

| **Subjects** | **Diarrhoea** | **Constipation** | **Bloating or abdominal distension** | **Nausea** | **Vomiting** | **Burping or regurgitation** | **Flatulence** | **Abdominal discomfort or pain** |
| --- | --- | --- | --- | --- | --- | --- | --- | --- |
| **Baseline** | | | | | | | | |
| **1** | 0 | 0 | 1 | 0 | 0 | 1 | 0 | 1 |
| **2** | 0 | 0 | 1 | 0 | 0 | 0 | 1 | 0 |
| **3** | 0 | 1 | 0 | 0 | 0 | 0 | 1 | 0 |
| **4** | 0 | 0 | 0 | 0 | 0 | 1 | 1 | 0 |
| **5** | 0 | 0 | 0 | 0 | 0 | 1 | 0 | 0 |
| **Day 28** | | | | | | | | |
| **1** | 0 | 0 | 0 | 0 | 0 | 0 | 0 | 0 |
| **2** | 0 | 0 | 0 | 0 | 0 | 0 | 0 | 0 |
| **3** | 0 | 1 | 1 | 0 | 0 | 0 | 0 | 0 |
| **4** | 0 | 0 | 0 | 0 | 0 | 1 | 1 | 1 |
| **5** | 0 | 0 | 0 | 0 | 0 | 1 | 1 | 1 |
| **Day 56** | | | | | | | | |
| **1** | 0 | 0 | 0 | 0 | 0 | 0 | 0 | 0 |
| **2** | 0 | 0 | 0 | 0 | 0 | 0 | 0 | 0 |
| **3** | 0 | 1 | 1 | 0 | 0 | 0 | 0 | 0 |
| **4** | 0 | 0 | 0 | 0 | 0 | 1 | 1 | 1 |
| **5** | 0 | 0 | 0 | 0 | 0 | 1 | 0 | 1 |

**Supplementary table 3** Tolerance (how well the subjects took) the RTU and powdered valine and isoleucine supplements were assessed on a scale of 1 to 5

Great = 5, Good = 4, OK = 3, Bad = 2, and Terrible = 1

| **Subjects** | **Valine powder** | **Valine liquid** | **Isoelucine powder** | **Isoleucine Liquid** |
| --- | --- | --- | --- | --- |
| **1** | 4 | 5 | 3 | 5 |
| **2** | 5 | 5 | 5 | 5 |
| **3** | 5 | 5 | 5 | 5 |
| **4** | 5 | 5 | 4 | 5 |
| **5** | 4 | 5 | 5 | 5 |
| **Total scores** | **23** | **25** | **22** | **25** |

**Supplementary Table 4.** Sodium intake from ready to use valine and isoleucine liquids compared with age-specific Reference Nutrient Intake (RNI) values. This comparison helps contextualize patient sodium intake relative to established dietary guidelines.

**Table 4.** Reference Nutrient intake of sodium (mg/day) by age, compared to patients’ sodium intake on RTU Val and Ile.

| **Subject** | **Patient age (years)** | **Reference Nutrient Intake of Sodium (mg/day) by age** | **Valine**  **1ml=50mg valine**  **Dose/day** | **Sodium intake mg/day*** | **Isoleucine**  **1ml=25mg isoleucine**  **Dose/day** | **Sodium Intake mg/day*** | **Total Sodium mg/day** | **% RNI Sodium** |
| --- | --- | --- | --- | --- | --- | --- | --- | --- |
| **1** | 12 | 1600 | 2ml | 5.2 | 6ml | 15.6 | 20.8 | 1.3 |
| **2** | 14 | 1600 | 2ml | 5.2 | 5ml | 13.0 | 18.2 | 1.1 |
| **3** | 3 | 500 | 3ml | 7.8 | 5ml | 13.0 | 20.8 | 4.2 |
| **4** | 8 | 1200 | 1ml | 2.6 | 12ml | 31.2 | 33.8 | 2.8 |
| **5** | 7 | 1200 | 2ml | 5.2 | 4ml | 10.4 | 15.6 | 1.3 |

*based on sodium 2.6 mg/mL, RNI reference nutrient intake, RTU ready to use
